# Synthesis and Characterization of EG/Au Composites via Thermal Exfoliation of Graphite Intercalation Compounds with Tetrachloroauric Acid

**DOI:** 10.3390/nano15171363

**Published:** 2025-09-04

**Authors:** Aleksandr D. Muravev, Andrei V. Ivanov, Vladimir A. Mukhanov, Boris A. Kulnitskiy, Natalia V. Maksimova, Victor V. Avdeev

**Affiliations:** 1 Chemistry Department, Lomonosov Moscow State University, Leninskie Gory 1-3, Moscow 119991, Russia; 2 Federal State Budgetary Institution “Technological Institute for Superhard and Novel Carbon Materials of National Research Centre «Kurchatov Institute»”, Centralnaya Str. 7a, Troitsk 108840, Russia

**Keywords:** carbon materials, graphite intercalation compound, thermal expansion, aurum nanoparticles, transmission electron microscopy

## Abstract

This study demonstrates a novel route to synthesize gold-decorated exfoliated graphite (EG) through graphite intercalation compounds (GICs) with tetrachloroauric acid (HAuCl_4_). We aimed to develop a scalable method for producing EG/Au composites with controlled nanoparticle morphology by investigating the effects of precursor chemistry and thermal expansion conditions. II-stage GIC–HAuCl_4_ (average gross-composition: C_23_HAuCl_4_; intercalate layer thickness d_i_ = 6.85 Å) was prepared via an exchange reaction of HAuCl_4_ with graphite nitrate. Interaction of this GIC with liquid methylamine yielded an occlusive complex, where methylamine-bound HAuCl_4_ occupies both interlayer and intercrystalline spaces in the graphite matrix. Methylamine treatment of GIC reduces the onset temperature of exfoliation by ≈100 °C and enhances the expansion efficiency, yielding EG with a low bulk density range of 4–6 g/L when processed at 900 °C in air or nitrogen. Thermal exfoliation of these GICs yielded EG decorated with gold nanoparticles, exhibiting a broad size distribution from a few nanometers to several hundred nanometers, as confirmed by electron microscopy. An X-ray diffraction analysis identified the coexistence of crystalline gold and hexagonal graphite phases, with no detectable impurity phases.

## 1. Introduction

Exfoliated graphite (EG) is a unique material produced by intercalation and subsequent thermal expansion of natural graphite [1]. This process results in the formation of thin, flake-like structures with a significantly increased surface area and porosity [2,3]. The crystal structure of EG is close to the structure of the original graphite, due to which composites based on it have high thermal stability, chemical inertness, and thermal and electrical conductivity along the graphene layers, together with high mechanical strength, elasticity, and flexibility [4,5]. Exfoliated graphite is widely used in the production of gaskets, seals [6,7,8], thermal management [9,10,11], and energy storage [12,13,14,15] systems due to its unique properties.

To impart new functional properties to exfoliated graphite, it can be doped with various metal-containing phases. The specific nature of these phases plays a critical role in determining the material’s performance for different applications. For instance, when developing magnetic sorbents for oil and hydrocarbon, EG is functionalized with ferro- or ferrimagnetic phases, which facilitate the collection and transportation of sorbate-saturated material [16,17,18,19]. For catalytic applications, EG/metal (EG/M) composites require not only a metal-containing phase of precise composition but also stringent control over the particle size, often necessitating dimensions below several tens of nanometers [20,21].

The following composites have already proven their effectiveness: EG/Fe in the decomposition of heavy metal salts [22], EG/Co in the Fischer–Tropsch processes [20], and EG/Pt for the formation of gas diffusion layers of fuel cells in chemical power sources [21]. The number of studies in which EG/Au or EG/Pt composites are used as catalysts is relatively small, since many researchers are focused on studying catalytic systems based on Au and Pt on more traditional supports, including activated carbon [23,24], carbon black [25,26], and “sibunit” [27]. This may be partly due to the current lack of scalable technology for producing such composites.

A common method for synthesizing EG/M composites involves depositing a metal-containing precursor onto expandable graphite, followed by thermal decomposition in an inert or reducing atmosphere to yield the desired metallic phase [28,29]. This approach enables precise control over the composition, size, morphology, and even shape of the resulting metal particles [30]. However, it is extremely low-tech, since the small mass of EG takes up a large volume. However, in our view, this method suffers from poor scalability due to the high volume-to-mass ratio of EG. EG exhibits an exceptionally low bulk density (≈2–4 g/L) [1], meaning any post-processing inevitably becomes a low-throughput operation.

An alternative approach enabling concurrent formation of the metal-containing phase during thermal expansion was previously developed. In this method, a mixture of expandable graphite, metal nitrates, and melamine undergoes thermal shock, producing EG decorated with iron, cobalt, and nickel particles of micron or submicron dimensions [31,32]. However, this system is unsuitable for investigating the factors controlling metal particle size. During air-based thermal treatment, the reduced metals rapidly oxidize to form oxides, while under inert atmospheres, they react with carbon to yield carbides.

Various ions, atoms, or molecules can be placed between the graphite layers. The compounds formed are called graphite intercalation compounds. The ions or atoms themselves, located between the graphite layers, are called intercalates. The amount of intercalate in graphite is determined by the stage number, which is numerically equal to the number of graphene layers between two adjacent intercalate layers [33,34].

GICs with gold chloride can be synthesized as either I-stage C_12.5_AuCl_3_ or II-stage C_25_AuCl_3_ compounds, characterized by an intercalated layer thickness (d_i_) range of 6.80–6.86 Å These compounds are prepared through a gas-phase synthesis approach using either single-zone or two-zone methods in sealed tubes. The synthesis requires elevated temperatures (200–300 °C) and prolonged reaction times ranging from 48 to 96 h [35,36,37]. The liquid-phase method enables synthesis of GICs with HAuCl_4_ under milder reaction conditions and with significantly reduced processing time compared to conventional approaches [38]. The synthesis is achieved by passing chlorine (acting as an oxidizer) through a molten mixture of tetrachloroauric acid hydrate and graphite, resulting in acid intercalation into the graphite matrix. A potentially more efficient approach involves an exchange reaction between HAuCl_4_ and pre-formed graphite nitrate. In this case, the graphite matrix already possesses a positive charge, potentially eliminating the need for additional oxidizers [21]. While previous work [21] confirms the successful preparation of GIC–H_2_PtCl_6_ via this exchange method, no published studies have documented its application for GIC–HAuCl_4_ synthesis.

This study investigates how synthesis conditions affect the structure of gold-containing thermally expanded graphite and the size distribution of gold particles derived from graphite intercalation compounds with HAuCl_4_.

## 2. Materials and Methods

### 2.1. Sample Preparation

To obtain GIC with gold chloride, the following reagents were used: natural flake graphite (particle size 250–300 µm, purity 99.7%), concentrated (98%) nitric acid, gold (purity 99.9%), nitric acid (solution 68–70%), hydrochloric acid (solution 35–38%), and isopropyl alcohol.

The synthesis of GIC with gold chloride was carried out by conducting an exchange interaction between IV-stage GIC–HNO_3_ (graphite nitrate) and HAuCl_4_. Graphite nitrate was obtained using a standard method during the interaction of graphite with concentrated 98% (fuming) HNO_3_ in the ratio for graphite/HNO_3_ of 1:0.5.

Gold was completely dissolved in aqua regia (3:1 mass ratio of 35–38% HCl to 68–70% HNO_3_) with gentle heating to 50–60 °C. The solution was then concentrated by evaporating excess solvent at 85–90 °C. The endpoint was determined by two visual indicators: near-complete cessation of vapor emission and a color evolution from initial yellow-orange to final dark red.

The resulting HAuCl_4_∙xH_2_O melt (in its residual crystallization water) was combined with freshly prepared graphite nitrate in a 1:5 mass ratio of graphite nitrate-to-HAuCl_4_∙xH_2_O. The reaction proceeded for 2 h at 85–90 °C with periodic stirring until constant mass was achieved. During this process, gradual mixture thickening and evolution of nitric acid vapor from the graphite intercalation compound as HAuCl_4_ replacing HNO_3_ were observed. After cooling to room temperature, the product was washed with excess isopropanol using a glass filter to remove residual acids, followed by drying in a furnace until constant mass was achieved.

GIC–HAuCl_4_ was saturated with ammonia and methylamine. For saturation, the following reagents were used: 25% aqueous ammonia solution, 38% aqueous methylamine solution, granulated potassium hydroxide, acetone, and liquid nitrogen.

The treatment of GIC–HAuCl_4_ with liquid ammonia (T_bp_ = −33 °C) and methylamine (T_bp_ = −6 °C) was carried out. Granulated KOH was added to a Wurtz flask with an aqueous solution of ammonia or amine. At the same time, gas began to release and passed through a column of alkali to dry out the water vapor captured from the solution. After passing through the column, the gas was condensed in a test tube placed in a thermos filled with acetone, which was pre-cooled with liquid nitrogen to a temperature of −50 ± 5 °C. After condensation of 3–4 mL amine (for 1 g of GIC), the gas supply to the test tube was stopped, and the resulting liquid phase was transferred to test tubes with GIC–HAuCl_4_.

After adding excess amine, the tube was sealed and kept at room temperature until the GIC was completely saturated with amine. After the holding period for at least 240 h, the tube was opened, and the excess amine evaporated at room temperature until the sample stopped losing weight. Next, the amine weight uptake was estimated, expressed as a mass percentage of the mass of the initially taken GIC.

The exfoliated graphite (EG) samples were prepared by thermal shock treatment of the GIC–HAuCl_4_ at temperatures of 700 °C or 900 °C, conducted either in ambient air using a muffle furnace or under nitrogen atmosphere in a custom quartz reactor.

### 2.2. Investigation Techniques

Phase compositions of all specimens were characterized by powder X-ray (XRD) diffraction using a Rigaku Ultima IV diffractometer (Tokyo, Japan), with CuK_α_ radiation (λK_α1_ = 1.5405 Å, λK_α2_ = 1.5443 Å).

Thermal decomposition behavior was investigated using a NETZSCH STA 449C Jupiter thermal analyzer (Selb, Germany). Samples (2–3 mg) were heated from 40 up to 400 °C at a rate of 5 °C/min under a nitrogen purge (100 mL/min).

The expansion of graphite particles was performed on an OLYMPUS BX51 optical microscope (Tokyo, Japan) with an OLYMPUS U-MSSP heated objective table (Tokyo, Japan) (temperature change rate of 50°/min). Filming was performed using an E3ISPM06300KPB color USB camera (Saint Petersburg, Russia). During editing, the video was sped up 5 times.

The bulk density (d_EG_, g/L) of gold-containing EG was calculated from the mass (m_EG_) and volume (V_EG_) of the powder:

d_EG_ = m_EG_/V_EG_

The error in determining the bulk density of EG was ±1 g/L.

The metal content was determined via a gravimetric analysis. The gold-containing EG samples (m_EG_ ~ 0.3 g) were heated in a ceramic crucible at 900 °C for 3 h in a muffle furnace to ensure complete combustion of the carbon matrix. The residual gold mass (m_Au_) was then measured, enabling calculation of the metal content in the expanded graphite:

ω_Au_ = m_Au_/m_EG_ × 100%

The bulk density of pure EG (d′_EG_, g/L) was recalculated using the Au content (ω_Au_) in the sample:

d′_EG_ = (1 − ω_Au_/100) × d_EG_

The morphology and elemental composition of the synthesized metal-containing EG samples were characterized by scanning electron microscopy (SEM) with energy-dispersive X-ray spectroscopy (EDX) using a TESCAN VEGA3 LMU instrument (Brno, Czech Republic).

The average metal particle size was determined using two complementary methods: a statistical analysis involving size measurements of 50 randomly selected particles across five representative micrographs per sample, and automated image processing, where the mean particle diameter was calculated from the total projected area of all particles in selected micrographs and manual particle counts, assuming spherical morphology.

Samples of EG with Au were examined by transmission electron microscopy (TEM) using a JEM-2010 transmission electron microscope (Tokyo, Japan).

EG was studied using the Raman scattering method using a Renishaw spectrometer (Wotton-under-Edge, the UK) with excitation by a laser, with a wavelength of 532 nm and laser power of 5% (0.8 mW, a spot diameter of 1 μm for objective ×50).

## 3. Results and Discussion

### 3.1. Synthesis of Graphite Intercalation Compounds with HAuCl_4_

GIC–HNO_3_ is a charged matrix [39] and is capable of entering into exchange interactions, such as with sulfuric acid and phosphoric acids, which are not capable of spontaneous intercalation without an external oxidizer [40], or hexachloroplatinic acid [21]. HAuCl_4_ is also a strong acid, so a similar reaction can be expected.

**Figure 1:**
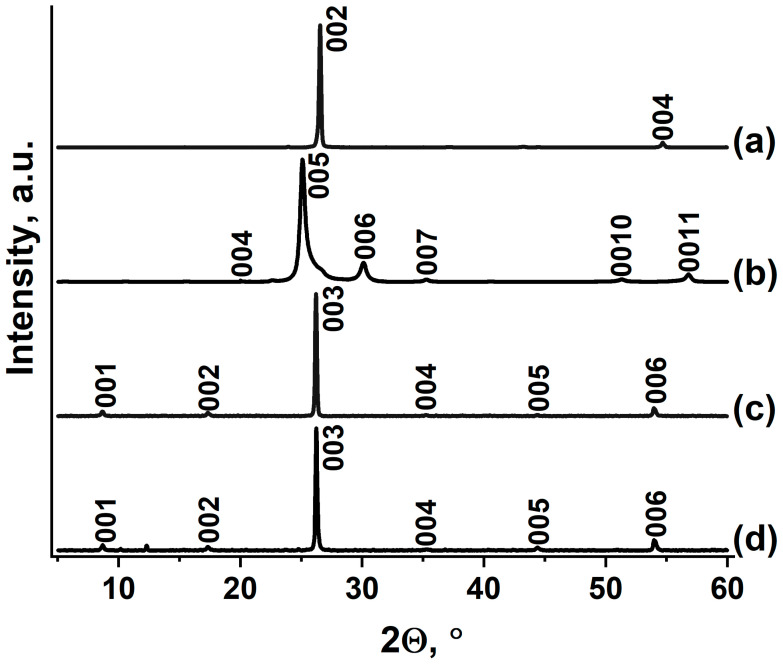
XRD patterns of (**a**) natural graphite flake, (**b**) GIC–HNO_3_, (**c**) GIC–HAuCl_4_, and (**d**) GIC–HAuCl_4_ treated by methylamine.

A graphite intercalation compound with HAuCl_4_ (GIC–HAuCl_4_) was prepared through an exchange reaction between graphite nitrate (GIC–HNO_3_) and tetrachloroauric acid. The GIC–HNO_3_ precursor was synthesized by treating natural graphite (Figure 1a) with fuming nitric acid. The identity period of GIC–HNO_3_ (I_c_ = 17.80 Å) was calculated based on an XRD analysis (Figure 1b), which confirmed the formation of stage IV. Metallic gold was first dissolved in aqua regia under gentle heating, with the solution subsequently concentrated through controlled evaporation until characteristic color changes were observed. The resulting HAuCl_4_∙xH_2_O melt was combined with GIC–HNO_3_, yielding a progressively thickening mixture as nitric acid was displaced from the graphite matrix.

During the reaction between GIC–HNO_3_ and HAuCl_4_∙xH_2_O, a known excess of melt HAuCl_4_∙xH_2_O is taken, i.e., theoretically, HAuCl_4_ should be sufficient to form a I-stage compound with the maximum degree of filling of the interlayer space of graphite, but this does not happen, apparently because the charge on the graphite matrix is insufficient for the intercalation process to lead to the formation of the I-stage, but is sufficient for the II-stage to form. In the paper devoted to the intercalation of H_2_PtCl_6_ during the exchange reaction with GIC–HNO_3_, it was also shown that only the II-stage GIC with H_2_PtCl_6_ is formed [21].

The final purification involved thorough washing with organic solvent to remove residual acids, followed by thermal drying to a constant mass. The average composition of the synthesized graphite intercalation compound was determined to be C_23_HAuCl_4_. In various experiments, the gross compositions of the obtained GICs varied from C_22_HAuCl_4_ to C_26_HAuCl_4_. This may be due to the existence of a fairly wide homogeneity region for this compound, which in principle is characteristic of acceptor-type GIC with strong acids.

The synthesized GIC–HAuCl_4_ exhibits an identity period of 10.20 Å, with an intercalated layer thickness (d_i_) of 6.85 Å, consistent with previously reported values [38]. These values correspond to the formation of II-stage GIC–HAuCl_4_. The XRD pattern (Figure 1c) demonstrates complete phase purity, showing no detectable reflections corresponding to either the initial graphite nitrate precursor, unreacted graphite, or non-intercalated gold species. These observations confirm the successful preparation of a homogeneous GIC through the exchange reaction between IV-stage GIC–HNO_3_ and HAuCl_4_.

It was found that GIC–HAuCl_4_ does not interact with liquid ammonia, unlike pure HAuCl_4_ [41]. While GIC–HAuCl_4_ remains inert toward liquid ammonia, it undergoes a selective reaction with liquid methylamine to form an occlusive complex with the stoichiometry {23C}HAuCl_4_‧2.7CH_3_NH_2_, corresponding to a 13.5 wt% methylamine mass gain. In order to distinguish “true” intercalated compounds from inclusion compounds in which the intercalate is located both in the interlayer and in the intercrystalline space of graphite obtained by processing the GIC with methylamine, we chose the following form {23C}HAuCl_4_‧2.7CH_3_NH_2_ to record the gross composition. This form of recording was proposed to be used by the authors of [42] when they studied the interaction of GIC–CuCl_2_ with ammonia.

Methylamine treatment of GIC–HAuCl_4_ does not result in the disappearance of the initial GIC diffraction peaks (Figure 1d), although their intensity decreases substantially, suggesting structural disordering. The appearance of two new unidentified reflections at 2Θ = 10.2 and 12.3° indicates the formation of either reaction products between deintercalated HAuCl_4_ and methylamine, or an intercalated complex within the graphite matrix. The reaction with methylamine results in partial extraction of HAuCl_4_ from the graphite matrix, while also forming a stable complex within the interlayer spaces near particle peripheries. Steric or kinetic limitations appear to restrict deeper intercalation, leaving some HAuCl_4_ unreacted in the bulk graphite, as evidenced by residual GIC reflections in the diffraction pattern. The room-temperature stability of the methylamine complex (despite methylamine’s low boiling point, −6 °C) suggests strong chemical interactions, likely coordination bonding between methylamine and the intercalated gold chloride species.

### 3.2. Thermal Decomposition of GIC–HAuCl_4_

**Figure 2:**
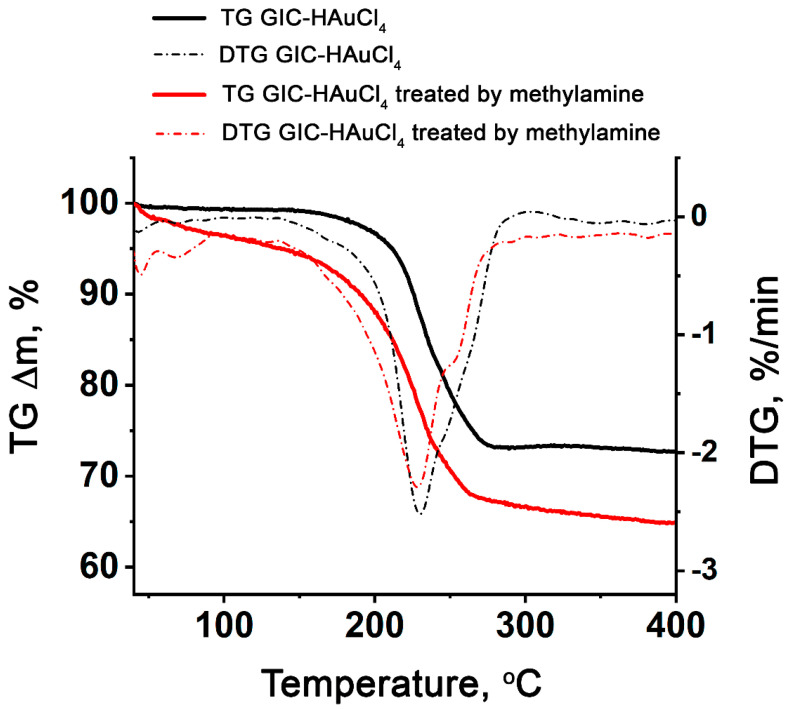
TG and DTG curves of GIC–HAuCl_4_ and GIC–HAuCl_4_ treated by methylamine.

The comparative analysis of thermogravimetric curves of GIC–HAuCl_4_ and GIC treated by methylamine reveals significantly lower thermal stability of the latter, which initiates decomposition at ≈50 °C, compared to 150 °C for the pristine GIC–HAuCl_4_ (Figure 2). The thermal decomposition profile of GIC–HAuCl_4_ exhibits a single distinct mass loss step, while GIC treated by methylamine demonstrates more complex degradation behavior.

According to [43], the thermal decomposition of the compound HAuCl_4_ occurs through concurrent dechlorination processes, with the HCl evolution occurring near 150 °C. The decomposition product, AuCl_3_, forms during the initial decomposition stage at 75–180 °C, consistent with the following reaction:

HAuCl_4(int)_ → AuCl_3(s)_ + HCl_(g)_

The secondary decomposition stage (180–235 °C) corresponds to decomposition of AuCl_3_:

AuCl_3(s)_ → AuCl_(s)_ + Cl_2(g)_

The final decomposition stage occurs between 235 and 320 °C, ultimately yielding elemental gold Au as the stable end product. This transformation is accurately represented by the reaction, consistent with previous observations [43]:

2AuCl_(s)_ → 2Au + Cl_2(g)_

The thermal analysis reveals concurrent decomposition processes occurring near 230 °C, including the decomposition of deintercalated gold chlorides, yielding gaseous HCl and Cl_2_ evolution, and breakdown of the GIC matrix. The GIC–HAuCl_4_–methylamine system displays additional thermal features on its TG/DTG patterns, including two low-temperature minima (<100 °C) corresponding to methylamine releasing from the complex, alongside the primary decomposition reactions (DTG minimum at 228 °C).

The thermogravimetric analysis reveals distinct mass losses of 27.3% for GIC–HAuCl_4_ and 35.2% for GIC–HAuCl_4_ treated by methylamine during decomposition. Assuming complete reduction to elemental gold on the graphite surface, the theoretical residuals from C_23_HAuCl_4_ and {23C}HAuCl_4_∙2.7CH_3_NH_2_ should both yield 23C + Au. This corresponds to calculated mass losses of GIC and treated GIC of 23.4% and 32.6%, respectively. The close agreement between experimental and theoretical values (with minor discrepancies attributable to methodological variations) confirms the proposed decomposition pathway.

To elucidate the deintercalation mechanism, we conducted in situ optical microscopy studies of GIC particle expansion during thermal treatment. Selected particles of both GIC–HAuCl_4_ and GIC treated by methylamine were deposited on a heating substrate. The samples were heated at a controlled rate of ≈50 °C/min, with temperature monitoring via a thermocouple integrated into the microscope stage. The entire thermal expansion process was recorded using the microscope’s digital imaging system (Video S1).

**Figure 3:**
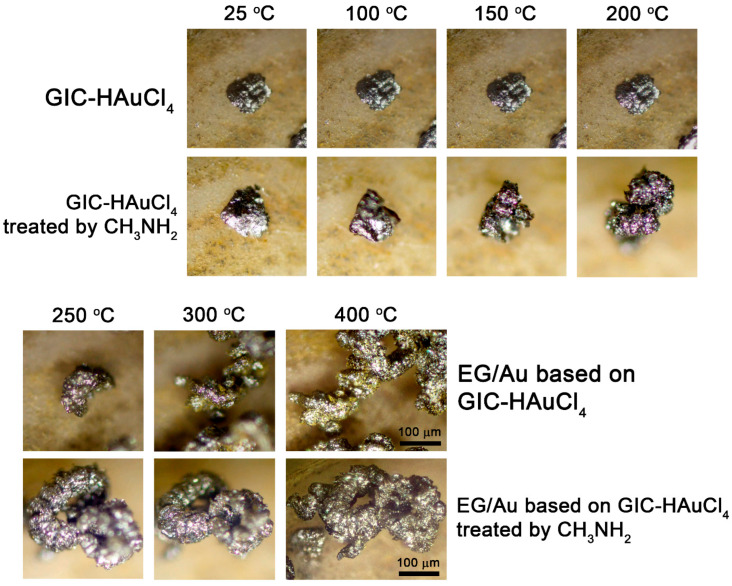
Optical images of GIC–HAuCl_4_ and GIC–HAuCl_4_ treated by methylamine after thermal treatment at different temperatures.

Optical microscopy observations (Figure 3) demonstrate distinct thermal expansion behaviors between the two materials. GIC–HAuCl_4_ treated by methylamine complex initiates expansion at ≈60–100 °C, while the GIC–HAuCl_4_ remains unchanged until reaching 190–200 °C. Complete expansion occurs at 280 °C, transforming the original flat GIC flakes into elongated, worm-like exfoliated graphite structures with visible metallic particles. These visual findings correlate well with thermogravimetric data. Methylamine treatment facilitates earlier GIC thermal expansion onset and enhances the overall expansion degree compared to the non-treated GIC.

### 3.3. Preparation of Exfoliated Graphite with Gold Particles

Rapid thermal treatment of acid-intercalated graphite induces violent intercalate evaporation and decomposition, generating substantial pressure within the graphite matrix [44]. During thermal decomposition, GIC–HAuCl_4_ and GIC–HAuCl_4_ treated by methylamine release HCl, Cl_2_, and melamine, which drive pronounced particle expansion and delamination. This process yields low-density powders consisting of elongated, worm-like particles of exfoliated graphite.

The resulting powder, composed of EG particles, exhibits a characteristically low bulk density. The EG samples were prepared through thermal expansion of GIC–HAuCl_4_ and GIC–HAuCl_4_ treated by methylamine at 700 °C and 900 °C in either air or nitrogen atmosphere. Three key experimental variables were investigated for their influence on material characteristics, namely the methylamine treatment, atmosphere, and expansion temperature. These parameters were hypothesized to affect both the structural properties of the EG matrix and the morphological distribution of surface gold nanoparticles. Table 1 presents the measured bulk densities of the EG samples and their corresponding gravimetrically determined Au contents.

**Figure 4:**
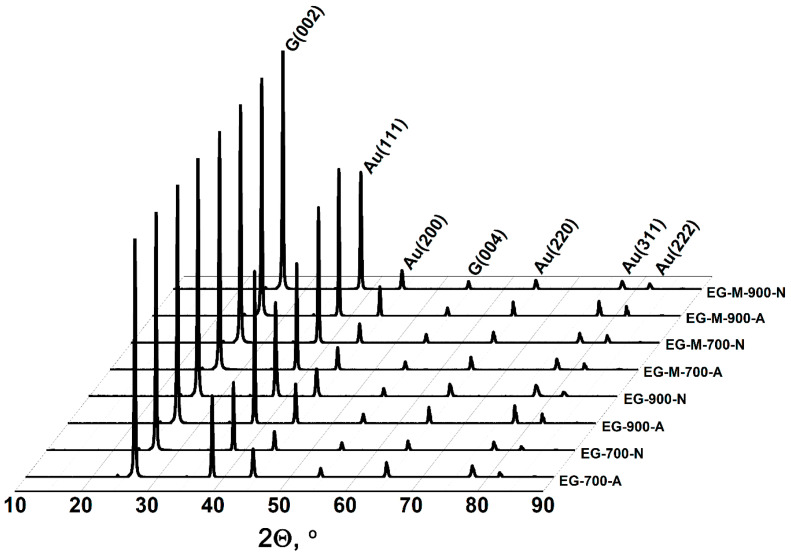
XRD patterns of gold-containing exfoliated graphite obtained under various processing conditions.

The experimental data demonstrate that lower EG bulk density is achieved under three key conditions: higher processing temperatures, which generate greater dispersive pressure; thermal expansion of GIC treated by methylamine, which enhances the dispersive pressure more significantly than temperature effects alone; and atmospheric processing in air rather than nitrogen, attributable not to the gas composition itself but to the superior thermal shock achievable in a muffle furnace compared to nitrogen-purged reactor systems. While the composition of EG remains largely unaffected by production conditions, oxidative processing in air results in partial carbon oxidation. This leads to a measurable increase in the gold-to-carbon ratio within the final product. The XRD analysis confirms that the phase composition of gold-containing EG remains consistent across all production conditions, exclusively comprising metallic gold with a face-centered cubic crystal structure and graphite (Figure 4).

**Figure 5:**
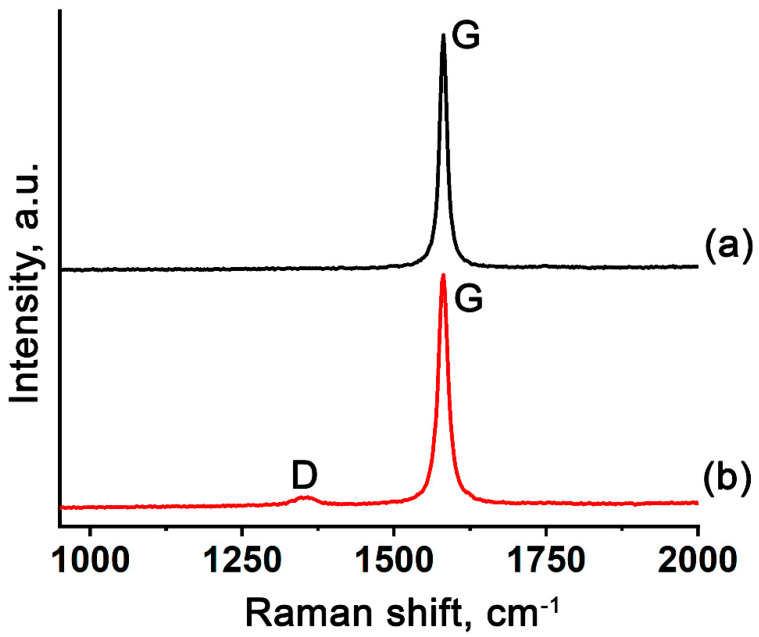
Raman spectra of gold-containing EG based on (**a**) GIC–HAuCl_4_ and (**b**) GIC–HAuCl_4_ treated by methylamine.

The Raman spectra of EG based on GIC–HAuCl_4_ and GIC treated by methylamine (Figure 5) show a prominent G-band, confirming high graphitic crystallinity in the EG-900-N and EG-M-900-N samples. EG based on GIC–HAuCl_4_ treated by methylamine additionally exhibits a distinct D-band, indicative of amorphous carbon formation [45,46]. This structural disorder likely results from thermal decomposition of methylamine’s hydrocarbon moieties under inert atmosphere conditions.

**Figure 6:**
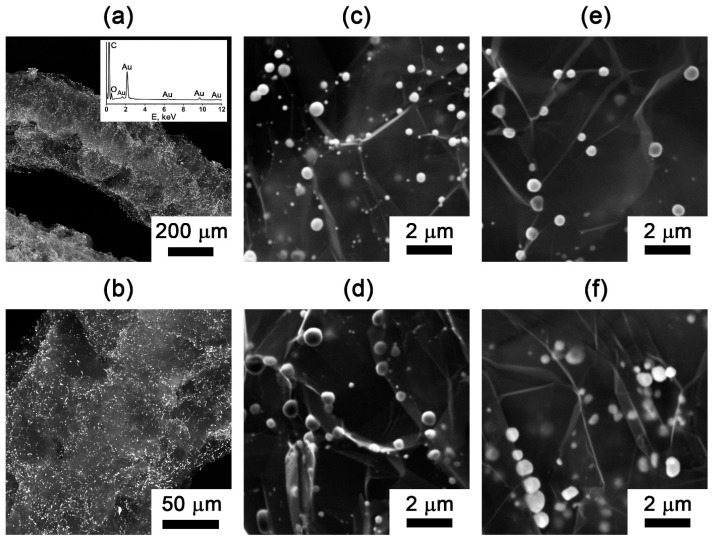
SEM images with an EDX analysis of the (**a**,**b**) gold-containing EG particles (EG-M-900-Air). SEM images of the gold particles on the surface of exfoliated graphite: (**c**) EG-M-900-Air; (**d**) EG-M-900-N; (**e**) EG-900-Air; (**f**) EG-900-N.

The SEM analysis reveals the characteristic worm-like morphology of EG particles (Figure 6a). Backscattered electron imaging demonstrates uniform metal distribution across the entire particle surface (Figure 6a,b). The gold particles are less than one micrometer in size (Figure 6c–f). The EDX analysis confirms the surface elemental composition consists exclusively of carbon, oxygen, and gold (Figure 6a). The observed oxygen signal originates from surface oxygen-containing functional groups, which is consistent with previously IR spectroscopy data and characteristic of all exfoliated graphite materials [47].

The average metal particle size was determined through a statistical analysis of representative SEM images, collected across all the EG samples. For quantitative assessment, one image from each set was subjected to the detailed image analysis, involving measurement of the total gold particle area, particle counting, and calculation of the mean spherical-equivalent diameter based on circular projection assumptions. The results for the two methods of determining the diameter are shown in Table 2. The two particle sizing methods demonstrate good correlation, with observed variations primarily attributable to the inherent polydispersity of the system. The broader size distribution obtained from the averaging method reflects this significant particle size dispersion.

Gold nanoparticles supported on exfoliated graphite exhibit submicrometer dimensions, with average particle sizes ranging from 300 to 500 nm (Figure 6c–f, Table 2). Our analysis reveals that the preparation atmosphere serves as the primary determinant of particle size, where nitrogen environments consistently yield larger Au particles compared to air. In contrast, both the methylamine treatment of the graphite intercalation compound and processing temperature range (700–900 °C) demonstrate comparatively minor influence on the final gold particle dimensions.

Based on the results of the conducted research and calculations, it turned out that the parameters we considered (methylamine treatment, atmosphere, and expansion temperature) have practically no effect on the size of the metal particles, but they do affect the degree of expansion of graphite and the bulk density of EG.

**Figure 7:**
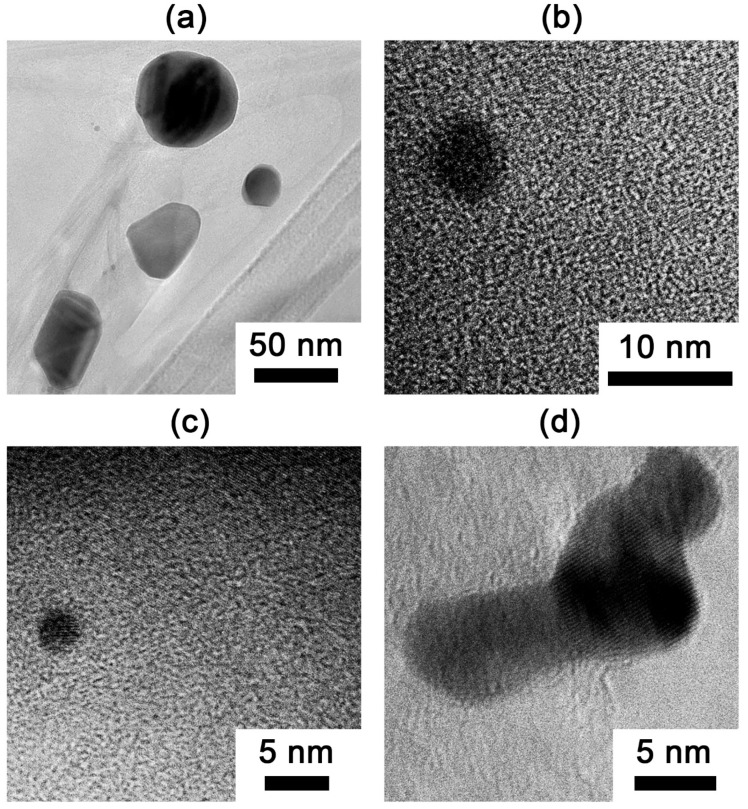
TEM images of gold nanoparticles: (**a**) nanoparticles with sizes in the tens of nanometers; (**b**–**d**) smaller nanoparticles (several nanometers in size).

Transmission electron microscopy was employed to characterize the gold nanoparticles dispersed within the graphite matrix of sample EG-M-900-A, which exhibited the lowest bulk density among all prepared samples (Figure 7). The TEM images reveal a size distribution of Au nanoparticles, ranging from tens of nanometers (Figure 7a) to several nanometers in diameter (Figure 7b–d).

Thus, this study demonstrates a versatile route for preparing gold-decorated exfoliated graphite with tunable nanoparticle sizes and distributions through optimization of the thermal conditions, reaction environment, and chemical precursors.

## 4. Conclusions

This work presents a robust synthetic strategy for fabricating exfoliated graphite decorated with gold nanoparticles via thermal treatment of a graphite intercalation compound with tetrachloroauric acid in air and nitrogen atmospheres. This work establishes, for the first time, a facile synthetic route to HAuCl_4_-intercalated graphite via exchange reaction between graphite nitrate and tetrachloroauric acid, bypassing the need for chlorine gas oxidation required by conventional gas-phase or liquid-phase methods. The resulting GIC–HAuCl_4_ can react with methylamine to form an intercalation complex, where coordinated amine–chloride species within the graphite’s matrix lower the onset temperature of exfoliation by ≈100 °C and enhance the expansion efficiency compared to untreated GIC.

Thermally exfoliated graphite composites exhibit gold nanoparticles with sizes ranging from a few nanometers to several hundred nanometers uniformly dispersed on low-density graphite matrices (6–19 g/L), with particle sizes governed primarily by the atmosphere of treatment rather than the processing temperature. The gold nanoparticles exhibit a polydisperse size distribution ranging from a few nanometers (<10 nm) to submicron dimensions (≈300–500 nm).

These fundamental insights into processing–structure relationships provide a rational design framework for engineering graphite-supported functional composites with tailored properties required in catalysis, energy storage, or functional coatings.

## Figures and Tables

**Table 1:** Characteristics of exfoliated graphite obtained from GIC–HAuCl_4_ under various processing conditions.

EG Sample	Initial GIC	Expansion Temperature, °C	Reaction Environment	d_EG_, g/L	ω_Au_, wt.%.	d′_EG_, g/L
EG-700-A	GIC–HAuCl_4_	700	Air	13	46.4	7
EG-700-N		700	N_2_	19	46.0	10
EG-900-A		900	Air	9	48.4	5
EG-900-N		900	N_2_	13	47.1	7
EG-M-700-A	GIC–HAuCl_4_ treated by methylamine	700	Air	5	47.4	3
EG-M-700-N		700	N_2_	8	46.6	4
EG-M-900-A		900	Air	4	48.1	2
EG-M-900-N		900	N_2_	6	47.5	3

**Table 2:** Results of determination of the average diameters of gold particles on EG.

Sample	Au Particle Size Measured Manually, nm	Au Particle Size Measured Through Area, nm
EG-700-A	380 ± 20	390 ± 40
EG-700-N	520 ± 30	420 ± 40
EG-900-A	370 ± 16	380 ± 40
EG-900-N	450 ± 19	450 ± 45
EG-M-700-A	360 ± 20	250 ± 25
EG-M-700-N	480 ± 20	440 ± 45
EG-M-900-A	380 ± 30	410 ± 40
EG-M-900-N	410 ± 20	310 ± 30

## Data Availability

Data will be made available on request.

## Supplementary Materials

The following supporting information can be downloaded at: https://www.mdpi.com/article/10.3390/nano15171363/s1, Video S1: Thermal expansion of intercalated graphite particles (GIC–HAuCl_4_ and GIC–HAuCl_4_ treated by methylamine).

## Abbreviations

The following abbreviations are used in this manuscript:

GIC	Graphite intercalation compound
EG	Exfoliated graphite
XRD	X-ray diffraction
SEM	Scanning electron microscopy
TEM	Transmission electron microscopy
EDX	Energy-dispersive X-ray spectroscopy
